# Reliability of prospective and retrospective maternal reports of prenatal experiences

**DOI:** 10.1186/s12884-022-05286-7

**Published:** 2022-12-27

**Authors:** Emily P. Rolan, Olivia Robertson, Nikolina Nonkovic, Kristine Marceau

**Affiliations:** 1 Department of Psychology, Michigan State University, 316 Physics Road, East Lansing, MI USA; 2 Department of Human Development and Family Science, Purdue University, 1202 W. State Street, West Lafayette, IN USA

**Keywords:** Maternal recall, Reliability of prenatal retrospective reports, Socioeconomic status, Prospective longitudinal, Prenatal stress

## Abstract

**Background:**

Extant perinatal research utilizes retrospective reports on the prenatal environment, but there are limited data on the validity of retrospective data compared with prospective data. The current study examined the reliability of birth mothers’ memory of prenatal stress and distress and perinatal risks at 6-months postpartum with maternal reports gathered across each trimester of pregnancy and explored whether recall varied with maternal socioeconomic status.

**Methods:**

Surveys were collected from 34 pregnant women (M age = 29.14, SD = 5.06 years, 83% non-Hispanic White) on stress, distress, and pregnancy complications at 12(T1), 26(T2), and 38(T3) weeks of pregnancy, and at 6-month post-partum asking the same questions but specifically about the pregnancy. Cohen’s kappa and Pearson’s correlations were used to investigate maternal recall at post-partum with prospective reports at T1, T2, T3 and an average score of T1, T2, and T3. Correlations were also examined separately for those with high and relatively lower socioeconomic status.

**Results:**

Birth mothers’ recall was generally reliable. Retrospective reports were most strongly related to prospective reports in T1 for perceived stress, T1 and T3 for anxiety symptoms and exposure to toxins, but T3 for depressive symptoms. Recall of pregnancy complications best reflected the average score across trimesters (rather than specific trimesters). Women with higher socioeconomic status better recalled prenatal (di)stress, but women with relatively lower socioeconomic status better recalled exposure to toxins.

**Conclusion:**

This study provides support for utilizing retrospective reports of maternal prenatal experiences at 6-months post-partum, with implications for interpretation of specific recalled phenotypes.

**Supplementary Information:**

The online version contains supplementary material available at 10.1186/s12884-022-05286-7.

## Background

Several risks or insults to the prenatal environment have been highlighted in the literature as being particularly problematic for fetal and child development, including maternal stress, anxiety and depression symptoms, pregnancy complications (e.g., maternal illness or infection), substance use (e.g., smoking during pregnancy), and toxins (e.g., lead or x-rays) [1–3]. Understanding the best and most feasible methods of data collection (e.g., medical records, prospective and retrospective maternal report, biological verification) across these different types of prenatal risk exposures remains a challenge. To this point, the literature has tended to focus on comparing the accuracy of medical records and retrospective maternal reports [4]. This literature has revealed that for some phenotypes (e.g., the severity of substance use during pregnancy), self-reported data may capture more actual risk exposure, whereas for other phenotypes (e.g., blood pressure) medical records provide more valid data [5]. Although limited in several known ways (see below) retrospective reports on prenatal events are often more feasible than collecting medical record or prospective reports during pregnancy, and so understanding the phenotypes, timing, and nuances of when and for what purposes retrospectively reported data may be more or less valid is an important undertaking in perinatal research [5–7].

Discrepancies between maternal retrospective report and medical record data could be due to limitations in mothers’ knowledge of prenatal events or to limitations in recall, although there are limited data on the validity of retrospective data compared with prospective data for risks. Limitations in recall can be based in inability to describe or inaccurate memory of events. Inaccuracy of recall might be a particularly important consideration for the prenatal period, to the extent to which that recall may be biased. For example, social desirability can distort memory recall for behaviors like prenatal substance use. As time progresses, pregnancy events are more likely to be under-reported; for example, women reported more prenatal complications 7 days post-partum than when re-questioned at 6 weeks and 6 months post-partum, although the salience of the event also matters: prenatal events that have longer-term impacts in terms of health issues are better recalled for longer periods of time [8]. However, not all relevant prenatal risks, namely prenatal stress and distress (i.e., anxiety and depressive symptoms), are present in medical record data. Although the literature includes both prospectively reported and retrospectively reported measures of prenatal stress and distress [9], to date, whether mothers accurately recall the levels of stress and distress they felt during pregnancy has not been investigated. The current study thus sought to compare prospective data gathered across each trimester of pregnancy with birth mothers’ memory of events at 6-months postpartum. Six months post-partum was chosen because this time point is often considered the end of post-partum maternal recovery, and prior studies commonly have used recall around 6-months post-partum to assess a wide variety of prenatal influences on infant and child development [10–12]. This study will provide needed data on the validity of retrospective reported data shortly after birth and could also illuminate bias in maternal reporting specific to recall.

### Prenatal stress and distress

Measures of prenatal psychosocial stress and distress are not systematically assessed during prenatal care in the United States, and thus do not appear in the medical record. At best, prescriptions for antidepressant or antianxiety medications may appear in the medical record. Sarangarm and colleagues (2012) conducted a study that suggests moderate to good agreement of maternal retrospective report of prescription data with medical records for antidepressant use [13]. However, there is growing literature that suggests antidepressants are overprescribed and, in many cases, their use does not align with psychiatric diagnosis criteria [14]. As such, the current best practice for measuring maternal psychological stress and distress during pregnancy is prospective reporting, which requires a longitudinal prospective design which may not be feasible for all studies.

Although the data are sparse, there is some evidence that retrospective reporting is moderately reliable for depression, but less-so for psychiatric medication use. For example, prospective and retrospective report agreement (measured via kappa coefficients) were small to moderate across measures (e.g., over the counter medication use, k = 0.05, non-psychotropic prescription use k = 0.28, psychotropic prescription use k = 0.52, depression symptoms k = 0.42) when comparing prospective ratings to retrospective recall at 6 months post-partum [12]. Newport and colleagues (2008) reported that recall error most often represented false negatives (compared to false positives), and participants were more likely to underreport events retrospectively [12]. As noted above, studies of psychosocial stress and distress measured via surveys have also been reported retrospectively, although the accuracy is assumed (but not tested) to be worse. The present study thus contributes sorely needed data on the validity of retrospective reporting in comparison to prospective reporting.

### Prenatal exposures and complications

For prenatal exposures and complications, pregnancy medical records and biological verification (e.g., of toxin and substance exposure) are the gold standard, although they are not without limitations. For example, the quality and completeness of medical records varies widely across participants in part due to differences in who fills out the records and when prenatal care began. Certified nurse-midwives keep more accurate records for maternal medical conditions, pregnancy complications, and intra-and postpartum events compared to physicians [6, 15, 16]. Further, some types of medical records such as birth certificates are not detailed or accurate enough to adequately measure some perinatal risks [6].

Thus, many studies, particularly those that studied the link between perinatal risks and later child outcomes, have used retrospective maternal reports in place of medical records because they can be more nuanced and are often more convenient to collect [7]. Notably, retrospective maternal reports correspond with medical records (e.g., infant and labor/delivery characteristics and smoking during pregnancy) [17], apart from specific medical information (i.e., blood pressure). Medical records and prenatal care records are preferred over retrospective recall for medical risks when available (e.g., bleeding, edema, weight gain) [5, 18]. However, there are instances where maternal retrospective report has been preferred over medical records, for example, when more nuanced assessment (e.g., the quantity/frequency of substance use) is of interest, or when women had late or little prenatal care [5]. When examined together, maternal but not birth record reports of smoking during pregnancy predicted infant birthweight [1, 19], suggesting that the key information about smoking during pregnancy that was relevant to birth weight was better captured via maternal report.

Understanding whether retrospective maternal reports reliably depict the prenatal environment is critical in determining the accuracy or types of bias that may be present (e.g., whether women tend to recall early or late pregnancy more readily) in the literature utilizing these methods of data collection. For example, social desirability can distort memory recall for behaviors like prenatal substance use. Further, in studies of retrospective reports, there is a tendency to over report positive memories, while negative memories fade more rapidly [20, 21]. Notably, when examining the validity of retrospective reports, comparisons of prenatal prospective and retrospective maternal reports have focused mostly on substance use (e.g., alcohol use, smoking during pregnancy, and illicit drug use) [22–25]. For tobacco or nicotine use, retrospective reports 14.5 years postpartum were accurate and reliable compared to prospective data gathered across pregnancy, especially when compared to prospective reports in the second and third trimester [25]. For alcohol use, prospective and retrospective reports were found to be moderately correlated (14 years apart, for average ounces per day and average ounces of alcohol per drinking day) [23]. However, another study found the retrospective report of prenatal alcohol use to be biased in comparison to prospective data; less than half of the women who reported alcohol consumption during pregnancy reported that they drank when asked 6 to 8 years postpartum [22]. Jacobson and colleagues (2002) [24] examined detailed smoking, alcohol and illicit drug use at each prenatal visit and 13 months postpartum and found moderate to strong correlations between reports (alcohol *r* = .60, cocaine *r* = .73, opiates *r* = .59, marijuana *r* = .49, and smoking *r* = .67). As evidenced by this literature, there is some disagreement on whether retrospective reports are biased compared to prospective reports, and existing published data largely consists of prenatal substance use. The current study extends this literature beyond the reliability of substance use, filling gaps in the literature by focusing on retrospective and prospective reports of maternal stress, depression, and anxiety symptoms, as well as pregnancy complications and exposure to toxins. Results will inform the validity and direction of bias of retrospective recall of these risks, which will help readers better interpret extant data and future data using retrospective reports of these prenatal risks.

### Socioeconomic status as potential moderator of recall

A small but growing literature also suggests that there may be socioeconomic disparities in validity of recall, which could affect interpretation of recalled perinatal phenotypes in some populations. For example, there was better recall for labor duration from women with more education [19, 26]. In a study of prenatal events, maternal report and medical record agreement did not largely depend on maternal characteristics, except for agreement between maternal reports and medical records for length of labor was better for women with more education and agreement for smoking during pregnancy was worse for those with higher socioeconomic status [16]. Maternal recall of infant birth weight has also been found to be better for women with higher socioeconomic status [27]. Thus, the current study also explored whether the reliability of maternal retrospective report when compared to prospective report depended on SES.

### Current study

In sum, there is lack data examining the validity of maternal retrospective reports regarding the prenatal environment beyond exposure to substance use. Retrospective reports can gather more nuanced prenatal data (e.g., depression and anxiety symptoms as opposed to diagnoses) and thus are a critical, and feasible tool for understanding the prenatal environment as it relates to physical and behavioral development, although data is needed to understand the utility and accuracy of these reports. Thus, the purpose of the current study was to (1) examine the reliability of birth mothers’ memory of perinatal risks and prenatal stress and distress (e.g., anxiety and depressive symptoms) at 6-months postpartum with maternal reports gathered across each trimester of pregnancy, and (2) explore whether recall depended on maternal SES. We hypothesized that (1) maternal postpartum recall would be reliable at 6-month with data gathered across pregnancy, and (2) that women with higher socioeconomic status would have more consistent recall.

## Methods

### Participants

Participants included 34 pregnant women (M age = 29.14, SD = 5.06 years). The sample is majority non-Hispanic White (83%), well-educated (56% with a college degree or higher), and ranks above the poverty line (75%; US DHHS, 2020; see Table 1 for full demographics). Infants’ gestational age at birth was on average 38.40 weeks (SD = 2.48, range = 29–41; 3 < 37 weeks, 3 at 37 weeks, 1 > 40 weeks). Women were recruited between July 2017 and October 2018, eligibility criteria for enrollment were that women (1) were less than 12 weeks pregnant (self-reported) and (2) lived within about a one-hour driving radius from Purdue University, in addition to (3) understanding the elements of informed consent, (4) understanding English at an 8th grade level, and (5) being over the age of 18 years. Participants completed questionnaires at 12 (M = 12.47, SD = 1.21; T1), 26 (M = 26.16, SD = 1.41; T2), 38 (M = 37.62, SD = 1.17; T3) weeks of pregnancy, and at 6-months post-partum (PP) the same items were asked about specifically *during pregnancy* (*n* = 30 with complete data). The overall retention rate was 95%, calculated relative to the sample of participants who completed the first assessment (*N* = 34). Three participants were lost to attrition (*N* = 2 left the study after T1; *N* = 1 left the study after T2) and were therefore excluded from the current study. The three participants that left the study were below the sample median for SES (see measures below). Two of the three participants reported some exposure to toxins and above the sample average pregnancy complications at T1. Further, all three participants were above the sample average for maternal stress, two participants had above sample average anxiety symptoms, and two had above sample average depressive symptoms. The study was approved by the Purdue University IRB (#1,704,019,124), and all participants provided informed consent. For further details on study procedures, see Marceau et al., 2021 [28].

**Table 1:** Demographic Information

	**Mean(SD)**	**Min-Max**
**Household Income**	$65,000 ($48,000)	0-$230,000
**Age at First Visit**	29.1 (5.1)	19.6-39.7
**Number of Children Residing in the Home at First Visit**	1.1 (1.2)	0-4
**Race**	**N (%)**	
White	25 (83.3)	
Black or African American	3 (10.0)	
Asian	1 (3.3)	
Latinx or Hispanic	1 (3.3)	
**Education**		
Less than high school degree	1 (2.9)	
High School degree/GED	11 (32.4)	
2-year college degree	3 (8.8)	
4-year college or university degree	11 (32.4)	
Graduate Degree	8 (23.5)	
**Employment Status**		
Unemployed/Student	12 (35.3)	
Part Time	5 (14.7)	
Full Time	17 (50.0)	
**Marital Status**		
Single, never married	6 (17.6)	
Married/Committed Living Together	28 (81.4)	
Separated/Divorced	0 (0.0)	

### Measures

#### Perceived maternal stress

Perceived stress during pregnancy was measured by maternal self-report on the perceived stress scale [29] at the T1, T2, T3, and PP assessments. Women were asked to report on 10 items that addressed their thoughts and feelings (e.g., “How often have you felt that you were unable to control the important things in your life?”) on a scale of 1 (*Never*) to 5 (*Very often*) over the previous month (T1, T2, T3). At the PP assessment, the prompt was changed to during their entire pregnancy (rather than over the previous month). The possible range of scores is 0–40. Internal consistency was acceptable, Cronbach’s α = T1: 0.91, T2: 0.88, T3: 0.84, and PP: 0.93.

#### Perceived maternal distress

Perceived distress during pregnancy was measured by maternal self-report of both anxiety and depression symptoms. The anxiety symptom score was created by summing 10-items from the Beck Anxiety Inventory (e.g., “did you have a time during your first trimester when you worried a lot more than most people would in your situation” and “how much were you bothered by heart pounding or racing”) [30] on a scaled of 1 (*Not at all*) to 4 (*Severely*), such that higher scores indicate greater anxiety symptoms. The possible ranges of scores are 10–40. Internal consistency was acceptable, Cronbach’s α = T1: 0.91, T2: 0.92, T3: 0.96, and PP: 0.92.

The depression symptom score was created by summing 13-items from the Beck Depression Inventory (e.g., “Was there every a time lasting 2 weeks or more when you lost interest in most things, like work, hobbies, or activities that usually give you pleasure?” and items related to feeling like a failure or feeling sad) [31], on a scale of 1 to 4, such that higher scores indicate greater depressive symptoms. The possible ranges of scores are 13–52. Internal consistency was acceptable, Cronbach’s α = T1: 0.83, T2: 0.89, T3: 0.90, and PP: 0.93.

#### Perinatal risk index

When used the Perinatal Risk Index (PRI) [11], an empirically based, standardized method for measuring overall perinatal risks weighted according to severity of obstetric complications based on the probability of causing harm to the fetus. The PRI was created on the premise that pregnancy complications must be considered on the whole for best empirical use and that the diverse type of complications require a weighting scale that considers the different risks to the infant [2, 3]. This coding scheme was judged to be highly relevant for our relatively small sample, as it considers the cumulative nature of perinatal risks as stressors that may impact the fetus during pregnancy and maximizes variability, allowing for relevant and meaningful (in terms of cumulative risks to the fetus) comparisons across prospective and retrospective data when single specific events are unlikely to be frequently endorsed. Thus, we created an index using methods from Knopik and colleagues (2016), which was largely determined using the McNeil-Sjöström obstetric complications scale [1–3, 11]. We focused specifically on two subscales for which we had sufficient variability in the data: *pregnancy complications* and *exposure to toxins*.

*Pregnancy complications* included questions regarding maternal age, number of prenatal visits, multiple pregnancies, irregular heartbeat, diminished fetal movements, bleeding from vagina, preeclampsia, high blood pressure or hypertension, weight gain or edema, obesity, low maternal weight gain, anemia, asthma, thyroid disorders, gestational diabetes, migraines or persistent headaches, epilepsy/seizure disorders, maternal infections (i.e., urinary tract infection, flu, upper respiratory tract infection, and sinus infection), fever or chills, vaginal discharge, nausea or vomiting, amniocentesis, and ultrasound. *Exposure to toxins* included questions regarding legal prescription drugs (i.e., “Please list all of the medication(s) that were prescribed by your doctor and why they were prescribed”), radiation (i.e., “Were you exposed to radiation”), X-Ray (i.e., “Were you exposed to x-rays”), lead (i.e., “Was the house or apartment that you lived in during your first trimester built before 1977?” and “Are you aware of any potential lead paint exposure in that house?”), and hazardous material (i.e., “Did your job involve any contact with hazardous materials like chemicals or asbestos?” and “Did you take precautions against exposure to hazardous materials at work?”).

Within each subscale, specific items were first weighted for potential risk to the fetus: 1 = not harmful or relevant, 2 = not likely harmful or relevant, 3 = potentially but not clearly harmful or relevant, 4 = potentially clearly harmful or relevant, 5 = potentially clearly greatly harmful/relevant, and 6 = very great harm to or deviation in offspring [3, 11]; See Table 2 for descriptive statistics). Then, within each subscale, two composite measures were formed, as per the scoring instructions in McNeil and Sjöström (1995). *Count scores* were created to examine the prevalence of experiencing a risk that posed potential (but not clear) harm/relevance to the fetus or higher (e.g., a score of 3 or higher), used to assess the number of risks present within each category. We also created *weighted risk scores* to examine the severity of risk weighted risk totals by summing risk scores that posed potential (but not clear) harm/relevance to the fetus or higher (e.g., a score of 3 or higher). A count score and weighted risk score were created for pregnancy complications and exposures to toxins at each time point (T1, T2, T3, and based on the PP where women reported on entire pregnancy). *Count scores* were used to describe data. However, to assess correspondence between retrospective and prospective recall, we used *weighted risk scores*, as these measures are most likely to reflect meaningful variation in perinatal risks that could affect the fetus and child development, and therefore are of higher interest.

**Table 2:** Descriptive Statistics of Key Study Variables

	During Pregnancy						Postnatal	
	12 weeks – T1 *N* = 34		26 weeks - T2 *N* = 32		38 weeks - T3 *N* = 31		6 months post-partum - PP *N* = 30	
	N	Mean(SD)	N	Mean(SD)	N	Mean(SD)	N	Mean(SD)
**Perceived Prenatal Stress**								
Perceived Stress Scale	34	15.12(7.57)	32	16.53(6.80)	31	15.42(6.83)	30	13.86(7.53)
**Perceived Prenatal Distress**								
Anxiety Symptoms	34	13.53(4.98)	32	12.31(4.58)	31	11.97(5.01)	30	12.97(5.05)
Depression Symptoms	34	19.21(5.16)	32	18.28(6.04)	31	17.58(5.57)	30	18.55(6.86)
**Pregnancy Risk Index (count scores)**								
Pregnancy Complications	34	1.69(1.02)	32	1.06(0.83)	31	1.59(1.07)	30	1.20(1.06)
Exposure to Toxins	34	0.66(0.84)	32	0.58(0.71)	31	0.69(0.82)	30	0.70(0.75)

For descriptive statistics on Pregnancy Risk Index weighted risk scores, please see Supplemental materials

#### Maternal socioeconomic status

Participants were asked to report demographics (e.g., family income, parental education, and maternal employment status), and complete financial need and financial deprivation questionnaires at T1. Financial need was assessed with a series of yes [1] or no (0) questions (e.g., taken an extra job to help meet basic living expenses) used to create a sum score, such that higher scores indicated more financial need [32, 33]. Financial deprivation was assessed with 6 items asking participants if they had enough month to afford items (e.g. “the place where I want to live” or “clothing that I should have”) on a scale from 1 (*strongly agree*) to 5 (*strongly disagree)*, an average score was created, such that higher scores indicate greater financial deprivation [32, 33]. Although much of the prior literature in this area [6, 23] has examined education as a proxy for socioeconomic resources, the link between indicators and specific resources is not well defined (e.g., educational attainment is not necessarily synonymous with increased access to resources) and there have been calls for more comprehensive measurement of socioeconomic resources [34]. Therefore, we created a socioeconomic status composite score, using a principal component analysis, integrating across indicators of access to resources: family income (continuously measured; M=$31,734.56/$96,462.50, SD=$21,025.29/$46,405.27 for relatively low/high socioeconomic status), maternal education (1 = less than high school degree, 2 = GED degree, 3 = high school degree, 4 = trade school, 5 = 2-year college degree, 6 = 4-yearcollege or university degree, and 7 = graduate program; M = 3.94/6.19, SD = 1.65/1.05 for relatively low/high socioeconomic status), maternal employment status (0 = not employed, 1 = employed part-time, 2 = employed full-time; ; M = 1.06/1.38, SD = 0.85/0.96 for relatively low/high socioeconomic status), financial deprivation (M = 2.52/1.42, SD = 0.81/0.52 for relatively low/high socioeconomic status), and financial need (M = 3.06/1.19, SD = 1.19/1.11 for relatively low/high socioeconomic status) [34]. The possible ranges of scores are: Maternal education = 1–7, maternal employment status = 0–2, financial deprivation = 0–12, financial need = 1–5. To examine differences in maternal recall of pregnancy complications by SES, we binned the socioeconomic status factor score at the median to look at correlations by high (above the socioeconomic status score median; *N* = 16) and relatively lower (below the socioeconomic status score median; *N* = 16) SES. For descriptive statistics of socioeconomic status indicators by high and relatively lower socioeconomic status, see Table 2. In general, women with relatively lower socioeconomic status reported somewhat higher levels of stress, distress, pregnancy complications, and exposures to toxins (see Supplemental Table S2).

### Data analysis

Agreement analyses (Cohen’s kappa and Pearson’s correlations) were used to investigate maternal recall at PP with T1, T2, T3 and an average score of T1, T2, and T3. Specifically, Cohen’s kappa was used for dichotomous variables (e.g., yes/no), whereas Pearson’s correlations were used for continuous variables. Recall was considered “very good” for k > 0.81, “good” for k > than.61, “moderate” for k > 0.41, “fair” for k > 0.21, and “poor” for k < 0.20. For correlations, recall was considered “strong” for *r* > .70, “moderate” for *r* > .40, and “weak” for *r* < .39. Although we interpret effect sizes, with a sample size of *n* = 30, we have sufficient power (0.80) to detect *r* correlations of ≥ 0.44 and at *p* < .05 and k coefficients of ≥ 0.65 (i.e., moderate effects) at *p* < .05. In analyses of socioeconomic status as potential moderator of recall, we examined correlations separately for those in high and relatively lower socioeconomic status but given the small sample sizes we only conducted descriptive analysis as an initial exploration.

## Results

### Maternal Recall of Perinatal Risks

#### Pregnancy complications

**Fig. 1:**
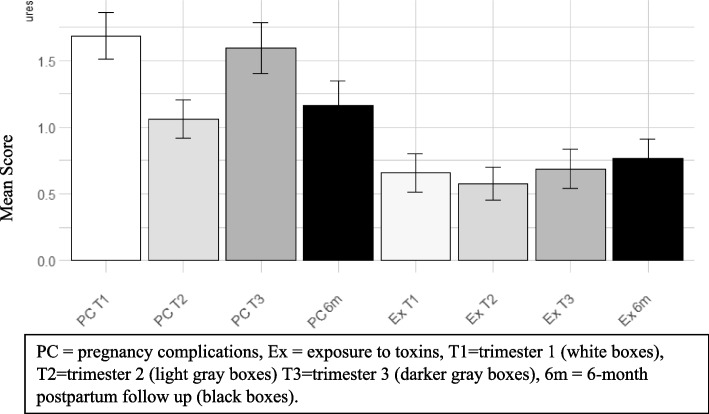
Pregnancy complications and exposure to toxins: Averages by trimester and post-partum. Note. The y-axis represents maternal mean scores on each perinatal index of risk (i.e., pregnancy complications and exposure to toxins). The x-axis represents the measure of risk at each trimester (T1, T2, T3) and the PP follow-up (6 m). Data show that for pregnancy complications, retrospective recall looked most similar to measures in the second trimester. For exposure to toxins, there does not appear to be much difference across trimester or post-partum

**Fig. 2:**
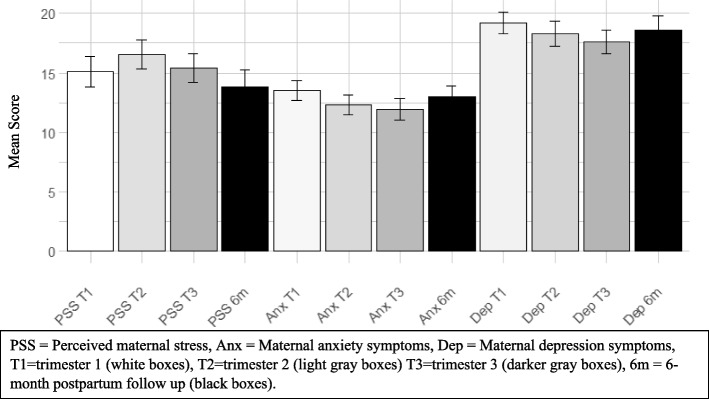
Stress measures: Averages by trimester and post-partum. Note. The y-axis represents maternal mean scores on each stress measure (i.e., perceived maternal stress, anxiety, and depression symptoms). The x-axis represents the stress measure at each trimester (T1, T2, T3) and the PP follow-up (6 m). Data show there are no real differences in the number of symptoms reported prenatally or retrospectively for perceived maternal stress, anxiety or depression symptoms

On average mothers reported experiencing 1.69 (T1), 1.06 (T2), 1.59 (T3), and 1.20 (PP) total pregnancy complications (e.g., on the *count* score) that posed potential (but not clear) harm/relevance to the fetus or higher (See Fig. 1). Prospective and retrospective maternal reports of pregnancy complications were moderately correlated for the weighted risk scores ranging from *r =* .38 to 0.43 (See Fig. 2; count score correlations were similar, please see supplemental material)

.

Across trimesters, the most prevalent complications (i.e., complications that occurred in more than 20% of the sample) at the risk threshold (a score of 3 on the McNeil-Sjöström scale described above, meaning possibly but not clearly harmful or relevant to the fetus) was low maternal weight gain (*N* = 32 for T1, *N* = 18 for T2, and *N* = 23 for T3). At PP, the most prevalent complications reported at the risk threshold were low maternal weight gain (*N* = 7) and hypertension (*N* = 6). When examining maternal recall of weight gain during pregnancy, we summed maternal reports of weight gain across T1, T2, and T3 (M = 27.35, SD = 20.78), and found that there was only moderate correlation, Pearson’s correlation *r* = .43, *p* = .08, with maternal reports of weight gained during their pregnancy PP (M = 22.48, SD = 14.92). For maternal recall of hypertension, agreement was poor in T1 (Cohen’s k = 0.12, *p* = .44), fair in T2 (Cohen’s k = 0.31, *p* = .06), and good in T3 (k = 0.68, *p* < .001). We also created an additional score of hypertension, such that 1 = did they ever report hypertension across T1, T2, or T3 and 0 = never reported hypertension during pregnancy and compared that to reports of hypertension PP. Agreement across T1, T2, and T3 with PP was moderately consistent (Cohen’s k = 0.53, *p* < .01).

#### Exposure to toxins

On average mothers reported experiencing 0.66 (T1), 0.58 (T2), 0.69 (T3), and 0.70 (PP) toxin exposures that posed potential (but not clear) harm/relevance to the fetus or higher (See Fig. 1). Prospective and retrospective maternal reports of pregnancy complications were moderately correlated for the weighted risk scores ranging from Pearson’s correlation *r* = .58 to 0.50 in trimester 1, 3, and for the average across trimesters (See Fig. 2; for count score correlations were similar except in T2, please see supplemental material, Figure S1). However, exposure to toxin weighted risk scores in T2 were not correlated with maternal reports at PP.

Across trimesters, the most prevalent (i.e., exposures that occurred in more than 20% of the sample) exposure to toxins were exposures to lead (*N* = 6 for T1, and *N* = 7 for T3) and hazardous materials (e.g., chemicals or asbestos; *N* = 12 for T1, *N* = 10 for T2, and *N* = 9 for T3). At PP, exposures to lead (*N* = 6) and hazardous materials (e.g., asbestos or chemicals; *N* = 12) remained the most prevalent toxin exposures. When examining maternal recall of exposure to lead, agreement of exposure to lead paint in the home or apartment was good for T1 (Cohen’s k = 0.75, *p* < .001), T2 (Cohen’s k = 0.81, *p* < .001), and T3 (Cohen’s k = 0.74, *p* < .001) when compared to PP. This item was contingent on residing in a home or apartment built before 1977, which was also reliably recalled: Cohen’s k = 0.87-0.93 across trimesters. Finally, when examining maternal recall of exposure hazardous material, this type of exposure was only reported in T1 and PP and the kappa coefficient suggested total disagreement (Cohen’s k=-0.04, p = .85). However, agreement for having to take precautions from hazardous material at work was moderately consistent for T1 (Cohen’s k = 0.50, *p* < .01) with PP and fairly consistent for T2 (Cohen’s k = 0.33, *p* = .06) and T3 (Cohen’s k = 0.26, *p* = .14) with PP.

#### Perceived maternal distress

**Fig. 3:**
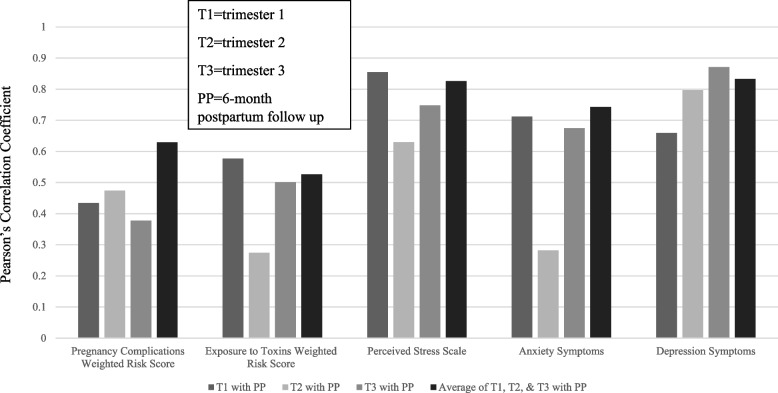
Pearson correlations of prospective and retrospective weighted risk scores with PP. Note. The y-axis represents the Pearson Correlation coefficient for the association between each trimester and the PP follow-up. The x-axis indicates which prenatal risk was being examined. The association with the PP follow-up was examined across T1, T2, T3, and an average across trimesters indicated by the varying colors of bars noted in the figure legend. All Pearson’s correlations, across prenatal risk and trimester, were statistically significant (*p* < .05). Statistical significance suggests good recall between the trimester and PP follow-up

Sample descriptive statistics are presented in Table 2. When looking across trimesters and PP, descriptive statistics do not appear to show a difference in the number of symptoms reported prenatally or PP (See Fig. 3). Further, prospective and retrospective maternal reports of perceived maternal distress were strongly correlated for anxiety symptoms at T1, T3, and the average score, ranging from 0.68 to 0.74 with PP (See Fig. 3). However, anxiety symptoms at T2 had a weak correlation with PP. Perceived maternal stress for depression symptoms were strongly correlated ranging from 0.66 to 0.93 across trimesters and the average score with PP (See Fig. 1).

#### Perceived maternal stress

Sample descriptive statistics are presented in Table 2. When looking across trimesters and PP, descriptive statistics do not appear to show a difference in the amount of symptoms reported prenatally or PP (See Fig. 2). Further, prospective and retrospective maternal reports of perceived maternal stress were highly correlated ranging from 0.62 to 0.86 across trimesters and the average score with PP (See Fig. 3).

### Socioeconomic status as potential moderator of Recall

**Fig. 4:**
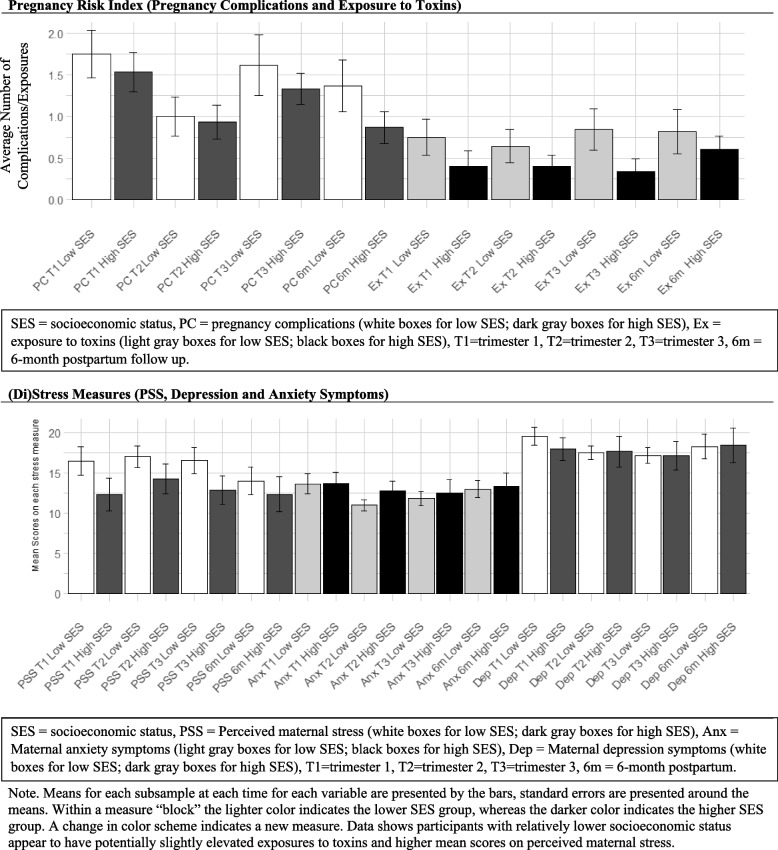
Descriptive statistics of key study variables by socioeconomic status. Note. Means for each subsample at each time for each variable are presented by the bars, standard errors are presented around the means. Within a measure “block” the lighter color indicates the lower SES group, whereas the darker color indicates the higher SES group. A change in color scheme indicates a new measure. Data shows participants with relatively lower socioeconomic status appear to have potentially slightly elevated exposures to toxins and higher mean scores on perceived maternal stress

Descriptive statistics for prenatal experiences are presented by SES in Fig. 4. Participants with relatively lower socioeconomic status appear to have an increased number of exposures to toxins, as well as potentially higher mean scores on perceived maternal stress.

#### Pregnancy complications

**Fig. 5:**
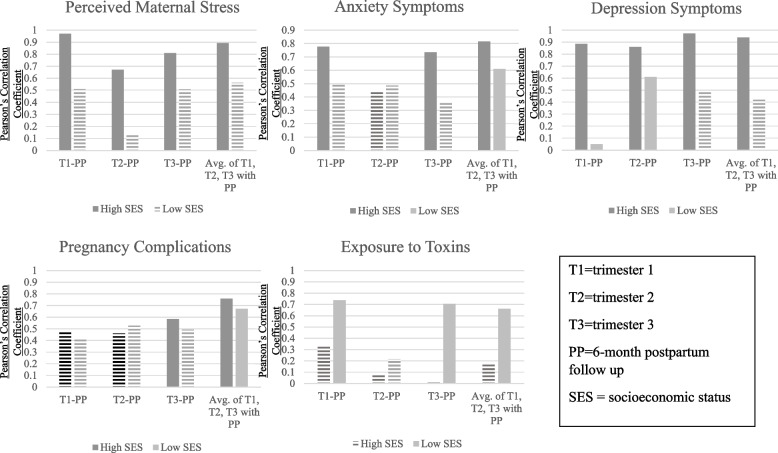
Pearson Correlation of perinatal risks by socioeconomic status. The y-axis represents the Pearson Correlation coefficient for the association between each trimester and the PP follow-up. The x-axis represents which trimester (T1, T2, T3 and an average across trimesters) is being examined in relation to the PP follow-up. Each trimester-PP follow-up association was examined by relatively high SES and low SES, indicated by dark grey bars (high SES) and light grey bars (low SES). Horizontal fill lines indicate (Pearson Correlation Coefficient, *p* > .05), solid columns indicate statistical significance (Pearson Correlation Coefficient, *p* < .05). Statistical significance suggests good recall between the trimester and PP follow-up, differences in SES can be used to interpret descriptive differences across levels of SES

When examining correlations of pregnancy complications by socioeconomic status, women with high socioeconomic status had better recall than women with relatively lower socioeconomic status, except for T2, for which the correlations with PP were lower in high socioeconomic status women than T1 or T3 (Fig. 5).

#### Exposure to toxins

Women with higher socioeconomic status had worse recall of exposure to toxins, with low correlations across trimesters with PP. Whereas, women with relatively lower socioeconomic status had better recall of exposures to toxins, given high correlations at PP with T1 and T3 (although not T2; see Fig. 5).

#### Perceived maternal distress

Maternal recall of anxiety symptoms was better for women with high socioeconomic status than those with relatively lower socioeconomic status (See Fig. 5). Similarly, for maternal recall of depression symptoms, women with high socioeconomic status had better recall with high correlations between all trimesters and PP, whereas those with low socioeconomic status appeared to have stronger recall of their depressive symptoms at T2 and T3 compared to T1.

#### Perceived maternal stress

For high socioeconomic status, women had better recall than women with relatively lower socioeconomic status (See Fig. 5).

## Discussion

The present study extended the literature examining correspondence between methods of assessing perinatal risks by comparing maternal recall of perceived stress, perinatal distress (i.e., anxiety and depressive symptoms), pregnancy complications, and exposure to toxins to prospective measures assessed near the end of each trimester. This analysis fills an important gap in the literature that reveals how women recall different types of perinatal adversities and will allow for better interpretation of studies using maternal retrospective recall. The number of women reporting specific prenatal complications adversities was lower at 6mo post-partum than they reported prospectively at T1 and T3, corroborating prior literature on increasing false negatives for pregnancy events over time (i.e., as time since the pregnancy increases) [21], however, the retrospective recall of pregnancy complications was largely consistent with T2 reports. Despite this difference in recall in terms of absolute severity of perinatal events (i.e., numbers of specific complications), exposures to toxins and mental health symptoms were reported in similar levels prospectively and retrospectively. Combined with moderate to high rank-order stability in overall levels of all of the prenatal risks, these findings suggest that retrospective recall can reliably capture between-person differences in overall (i.e., pregnancy-average) levels of perinatal risks. Further, across all measures, correlations of recall at 6-months postpartum with the computed average levels across pregnancy were nearly as high or higher than any given trimester. This finding suggests that retrospective recall can reliably be interpreted as a pregnancy-average level for medical and psychosocial prenatal risks.

We also supported prior literature in suggesting that women with higher socioeconomic status generally have better recall than women with relatively lower socioeconomic status [16, 19, 25], except for exposures to toxins which women with relatively lower socioeconomic status recalled with higher accuracy. These findings are new and should be considered preliminary as they were not tested using a formal statistical comparison due to the sample size, but the pattern of findings - that women with relatively lower socioeconomic status may potentially have better recall of exposure to toxins is similar to a prior finding that women with lower socioeconomic status were better at recalling smoking during pregnancy [16]. Notably, it may be that recall for exposure to toxins was potentially higher for women with relatively lower socioeconomic status due to differential risk exposure, as women in this group have greater exposure to toxins and thus increased awareness or opportunities to recall exposures. These health exposures may also be particularly salient for women who perceive more negative consequences [8], as may be the case for women living in lower socioeconomic conditions where they may have less ability to avoid such exposures despite widespread knowledge of toxin exposures like lead on the developing child. It may also be that women with relatively higher socioeconomic status under report substance use exposure, as they might be more aware of negative consequences or the social unacceptability of the behavior. Future research, using larger samples with more socioeconomic diversity, is needed to confirm these findings with formal statistical comparisons and to test for processes through which this association may occur.

We found that women were quite adept at recalling their levels of stress and distress experienced during pregnancy at 6-months postpartum. However, our data suggests that women may be thinking of primarily the first trimester when recalling levels of stress, the first and last trimester (not the second trimester) when recalling levels of anxiety, and were recalling especially the third trimester, but generally later pregnancy when recalling their levels of depressive symptoms. Women were only somewhat adept at recalling their pregnancy complications and exposures to toxins (with moderate correlations). This finding is similar to studies comparing retrospective reports and medical record reports, finding lower agreement for medically-relevant risks [5, 17, 18]. Like measures of perceived anxiety and stress, women seemed to be thinking about/better recalling the first and third trimesters when recalling their exposures to toxins. In contrast, maternal recall of pregnancy complications reflected the pregnancy as a whole, rather than specific periods of the pregnancy. This may indicate that women more reliably identify whether a pregnancy complication happened at all, rather than being anchored to a particular period (e.g., early or late pregnancy) as found when recalling feelings of stress and distress.

Notably, the types of pregnancy complications experienced by our sample were relatively mild (low weight gain and hypertension, as opposed to problems with fetal anatomy or major medical procedures that may be more emotionally salient. The literature on emotional arousal in short-and long-term memory suggests that emotional arousal may enhance long term memory [35]. In particular, a growing body of literature suggests that sex hormones can influence women’s recall of stressful or emotional events (e.g., progesterone), such that increased progesterone is linked to more sensitivity to sources of threat or danger [36]. Notably, progesterone is higher in pregnant women and increases across pregnancy [28]. For these reasons, pregnant women may be more likely to attach details, such as timing of events, to prenatal stress and distress (i.e., events with greater emotional arousal), as opposed to pregnancy complications (which may vary in their level of threat or danger, as perceived by the reporter). However, further work examining the interaction of sex hormones with birth mothers’ reports on the level of stress surrounding events (e.g., pregnancy complications versus depressive symptoms) is necessary to confirm this proposed line of reasoning.

### Strengths and limitations

The repeated measures of perceived stress and distress, and pregnancy complications and exposures to toxins (weighted for the potential for risk to the fetus) at tight gestational age windows near the end of each trimester and again at 6-months postpartum were strengths of the study. Another strength of the current study is the focus on relevant prenatal risks that are not necessarily present in the medical record, like prenatal stress and distress (i.e., anxiety and depressive symptoms. The largest limitations were that the study was limited in sample size and in the sociodemographic composition of the sample (which was primarily White, educated women). The small sample size means that our standard errors are quite large, increasing risk of type II error and potentially unstable estimates of effect sizes. Further, aspects of attrition in the current sample may further inflate these issues. Effects should thus be considered preliminary and are best used as pilot data to generate expected effect sizes in future research. These women are not likely facing the types of stress and adversity many marginalized or minoritized women experience, and thus the generalizability of these findings to at-risk samples is limited. Our reports of relatively lower socioeconomic status are likely not capturing the risk that may be found in a more diverse sample (e.g., increased anxiety or exposure to toxins). Due to the small sample size and lack of diversity, future studies with larger samples experiencing more adversity and complications are needed to confirm and extend these findings. Finally, stemming from our small sample size, we were unable to examine specific prenatal risks, which may vary in recall. Despite these limitations, our findings are applicable to interpreting many extant studies that suffer similar limitations in generalizability.

## Conclusion

These findings contribute three main ideas to the literature. One, we found preliminary evidence that women may anchor their thoughts primarily in early pregnancy when re-calling stress, early and late pregnancy when recalling anxiety and exposures to toxins, and later pregnancy when recalling depressive symptoms. Two, we also show that even with only relatively lower SES, recall bias differs. Specifically, the accuracy of maternal reports was worse for stress and anxiety among women with relatively lower SES, but better for exposure to toxins. Women with relatively lower socioeconomic status anchored their recall of depressive symptoms in later pregnancy, rather than pregnancy as a whole. Three, recall of pregnancy complications, although only moderately accurate, did not vary by SES. However, based on the relatively lower reliability of recall for pregnancy complications, much of the inconsistency between medical record vs. retrospective self-report in medical risks likely reflects to some degree reduced accuracy of retrospective report. Thus, our findings clarify that extant findings in the literature that uses primarily White and educated samples (of which there are many) that use retrospective recall of prenatal stress and distress are likely not limited to a great extent by recall bias but are somewhat limited by recall bias for medical risks. However, researchers examining increasingly at-risk populations may reconsider the use of retrospective reports for prenatal (di)stress). Importantly, studies using retrospective maternal reports may need to be replicated in populations of marginalized or minoritized women and future work should incorporate socioeconomic status into their study design (e.g., testing to see whether findings differ by indicators of socioeconomic status).

## Supplementary Information

**Additional file 1:** **Table S1.** Demographic Information by High and Relatively Lower SES. **Table S2.** DescriptiveStatistics of Key Study Variables by High and Relatively Lower socioeconomic status. **Table S3.** Descriptive Statistics of Weighted Risk Scores for the Perinatal Risk Index. **Figure S1.** Pearson correlations of prospective and retrospective count scores.

## Data Availability

The dataset used and/or analyzed during the current study are available from the corresponding author on reasonable request.

## Abbreviations

T1: First Trimester
T2: Second Trimester
T3: Third Trimester
PP: Postpartum
SES: Socioeconomic Status
PRI: Perinatal Risk Index
