# Supplementary material for: Reliability of prospective and retrospective maternal reports of prenatal experiences
Source: BMC Pregnancy Childbirth. 2022 Dec 27;22:968. doi: 10.1186/s12884-022-05286-7 (PMC9793511; doi:10.1186/s12884-022-05286-7)
Supplement: Supplementary file 1 — Additional file 1: Table S1. Demographic Information by High and Relatively Lower SES. Table S2. DescriptiveStatistics of Key Study Variables by High and Relatively Lower socioeconomic status. Table S3. Descriptive Statistics of Weighted Risk Scores for the Perinatal Risk Index. Figure S1. Pearson correlations of prospective and retrospective count scores. [file 12884_2022_5286_MOESM1_ESM.docx]

Supplemental Material

| Table S1. Demographic Information by High and Relatively Lower SES | | | |  |  |
| --- | --- | --- | --- | --- | --- |
|  |  | **High SES** | | **Relatively Lower SES** | |
|  |  | **Mean(SD)** | **Min-Max** | **Mean(SD)** | **Min-Max** |
| **Household Income** | | $96,462.50($46,405.27) | $56,000-$230,000 | $31,734.56($21,025.29) | $0-$63,000 |
| **Age at first visit** | | 30.27(4.78) | 21.59-39.71 | 28.17(4.91) | 19.53-34.98 |
| **Number of Children** | | 1.06(1.29) | 0-4 | 1.44(1.26) | 0-4 |
|  |  |  |  |  |  |
| **Race^a^** | | **N (%)** |  | **N (%)** |  |
|  | White | 13(76.5) |  | 10(62.5) |  |
|  | Black or African American | 2(11.8) |  | 1(6.3) |  |
|  | Asian | 1(5.9) |  | -- |  |
|  | Latinx or Hispanic | 1(5.9) |  | -- |  |
| **Education** | |  |  |  |  |
|  | Less than high school degree | -- |  | 1(6.3) |  |
|  | High School degree/GED | 1(6.3) |  | 9(56.3) |  |
|  | 2-year college degree | 1(6.3) |  | 2(12.5) |  |
|  | 4-year college or university degree | 7(43.8) |  | 3(18.8) |  |
|  | Graduate Degree | 7(43.8) |  | 1(6.3) |  |
| **Employment Status** | |  |  |  |  |
|  | Unemployed/Student | 5(31.3) |  | 5(31.3) |  |
|  | Part Time | -- |  | 5(31.3) |  |
|  | Full Time | 11(68.8) |  | 6(37.5) |  |
| **Marital Status** | |  |  |  |  |
|  | Single, never married | -- |  | 6(37.5) |  |
|  | Married/Committed Living Together | 16(100) |  | 10(62.6) |  |

Note. 2 white participants had missing socioeconomic status data and therefore were not included in the table.

| Table S2. Descriptive Statistics of Key Study Variables by High and Relatively Lower socioeconomic status | | | | | | | | |
| --- | --- | --- | --- | --- | --- | --- | --- | --- |
|  | **High SES** | | | | **Relatively Lower SES** | | | |
|  | 12 weeks – T1 | 26 weeks - T2 | 38 weeks - T3 | 6 months post-partum - PP | 12 weeks – T1 | 26 weeks - T2 | 38 weeks - T3 | 6 months post-partum - PP |
|  | Mean(SD) | Mean(SD) | Mean(SD) | Mean(SD) | Mean(SD) | Mean(SD) | Mean(SD) | Mean(SD) |
| **Perceived Prenatal Stress** |  |  |  |  |  |  |  |  |
| Perceived Stress Scale | 12.81(7.89) | 14.81(7.24) | 13.44(7.03) | 12.33(8.42) | 16.50(6.94) | 17.00(5.02) | 16.68(5.73) | 14.00(5.73) |
| **Perceived Prenatal Distress** |  |  |  |  |  |  |  |  |
| Anxiety Symptoms | 13.88(5.24) | 12.63(4.40) | 12.31(6.45) | 13.33(6.35) | 13.63(5.03) | 11.00(2.54) | 11.85(3.16) | 13.00(3.58) |
| Depression Symptoms | 18.19(5.26) | 18.06(7.21) | 17.38(6.64) | 18.47(8.24) | 19.72(4.27) | 17.50(3.11) | 17.70(3.89) | 18.27(5.08) |
| **Pregnancy Risk Index** |  |  |  |  |  |  |  |  |
| Pregnancy Complications | 3.06(0.99) | 8.59(0.83) | 8.83(0.87) | 21.53(1.13) | 10.40(1.13) | 11.07(0.88) | 11.13(1.33) | 23.27(1.03) |
| Exposure to Toxins | 0.59(0.87) | 0.59(0.71) | 0.53(0.80) | 0.65(0.70) | 0.75(0.86) | 0.64(0.74) | 0.85(0.90) | 0.82(0.87) |

Note. SES = socioeconomic status.

| Table S3. Descriptive Statistics of Weighted Risk Scores for the Perinatal Risk Index | | | | | | | | |
| --- | --- | --- | --- | --- | --- | --- | --- | --- |
|  | During Pregnancy | | | | | | Postnatal | |
|  | 12 weeks – T1  N=34 | | 26 weeks - T2 N=32 | | 38 weeks - T3  N=31 | | 6 months post-partum - T5  N=30 | |
| Measure | N | Mean(SD) | N | Mean(SD) | N | Mean(SD) | N | Mean(SD) |
| **Pregnancy Risk Index** |  |  |  |  |  |  |  |  |
| Pregnancy Complications | 34 | 5.79(3.27) | 32 | 3.50(2.76) | 31 | 5.00(3.78) | 30 | 4.20(3.61) |
| Exposure to Toxins | 34 | 2.03(2.62) | 32 | 1.73(2.13) | 31 | 2.09(2.557) | 30 | 2.13(2.36) |

Note. Pregnancy Risk Index scores are the weighted risk scores.

Figure S1. Pearson correlations of prospective and retrospective count scores.

Pearson’s Correlation Coefficient

Note. T1=first trimester, T2=second trimester, T3=third trimester, PP=6-month postpartum follow-up. The y-axis represents the Pearson Correlation coefficient for the association between each trimester and the PP follow-up. The x-axis indicates which prenatal count score was being examined. The association with the PP follow-up was examined across T1, T2, T3 and an average across trimesters indicated by the varying colors of bars noted in the figure legend. Correlations for prenatal substance use is not available in the first trimester because data were not collected due to technical errors. All Pearson correlations were statistically significant (p<.05), except for exposure to toxins in the second trimester with the 6-month postpartum follow-up.
